# Changes of the patient management in dentistry during the pandemic caused by the SARS-Coronavirus 2—initial perspectives of a clinic of operative dentistry in Europe

**DOI:** 10.1007/s00784-020-03351-z

**Published:** 2020-05-29

**Authors:** Stefan Rupf, Matthias Hannig

**Affiliations:** Clinic of Operative Dentistry, Periodontology and Preventive Dentistry, Saarland University, Kirrberger Str. 100, Building 73, D-66421 Homburg/Saar, Germany

## Summary

This Letter to the Editor presents a concept for the patient group-related dental treatment during the coronavirus disease 2019 (COVID-19) pandemic. This principle has been developed based on the cooperation of teams of oral health care professionals combined with a rotation system for use of dental units.

## Background

The outbreak of the severe acute respiratory syndrome coronavirus 2 (SARS-CoV-2) in China at the end of 2019 has progressed into a pandemic [1, 2]. While it has successful managed to limit the number of new infections and deaths in China [3], the world is probably still facing the peak of new infections and severe causes of COVID-19 [4].

In Germany, more than 0.15% of the population had been tested positive for SARS-CoV-2 at the middle of April [5]. In Europe, the situation in the health care systems is already extremely challenging in some countries, in particular in Italy [6]. In other countries, such as Austria and Germany, there is currently still sufficient capacity to treat severe cases and the number of deaths caused by SARS-CoV-2 is still low [5, 7].

The pandemic also creates new challenges for dentistry. There are many therapeutic procedures that lead to the release of aerosols [8, 9]. It is currently impossible to assess the risk to dental professionals with any degree of confidence because rapid and reliable tests for the salivary diagnostic for SARS-CoV-2 are not available [8].

In Germany, Institute of German Dentists, in accordance with the Robert Koch Institute, recommends comprehensive measures to protect staff and to limit dental care to emergencies [10]. Moreover, elective treatments should be avoided as far as possible in order to interrupt transmission routes and preserve resources such as protective equipment. Interventions should be postponed to post-pandemic time, and more attention should be paid to the use of electronic patient counselling resources by phone or video [10].

## Point of view

In this statement, we describe the principles for the patient management of the Clinic of Operative Dentistry, Periodontology and Preventive Dentistry of the Saarland Medical Centre. We considered experiences of Chinese dental clinics [11] and the current German [10] regulations on patient management.

This concept might be transferable to other dental clinics as well as in general to other dental offices.Our recommendation is based on:

- the cooperation of several teams of dental professionals and
- a rotation system for the treatment rooms resp. dental units.

An important premise is that patients have to contact the treatment centre and are already assigned by telephone to patient groups described below (triage). Patients are then called into the clinic on the next possible appointment. Patients who appear on their own initiative are provided with a fixed appointment at the earliest possible time. Only one patient is present in the treatment section at any time. If a separable and safely sanitizable waiting zone is available, the delayed appointment of a second patient can be considered. In the Clinic of Operative Dentistry, six dentists supported by six dental assistants usually treat 35–70 patients daily, in addition to their responsibilities in the undergraduate teaching of dental students. The therapeutic spectrum covers all areas of dentistry with the exception of orthodontics and maxillofacial surgery. There are six dental units available for the treatment of patients in separate rooms. The facilities for the training of undergraduates are currently not used. Currently, only cases of emergency are treated and the number of patients is reduced to less than 10 patients per day.

In addition to the above-mentioned general recommendations, two important questions emerge in the current situation:

I. How can the management of the different patient groups be organized safely?
II. How can staff and treatment rooms resp. dental units be used appropriately?

As long as no rapid and reliable test system is available that offers a high probability of excluding an infection, patients are divided into three groups:

A. patients who present typical symptoms of colds, flu-like infections or influenza, such as cough, rhinitis or fever, including COVID-19 and SARS-CoV-2 positive;
B. patients without respiratory signs whose infection status is unknown [12]; and,
C. highly vulnerable corona-negative patients in whom COVID-19 is associated with a high risk of severe course of disease [13].

In the management of patients in groups A and B, the protection of the staff is of primary concern. For patients in group C, the protection of the patient from infection by the staff is crucial as well. Based on our previous experience in the situation of a still continuing spread of SARS-CoV-2, patients in group A are single cases, but the time and material effort for their treatment is very high. Patients in group B appear more often in our clinic, while patients in group C do not need to be treated as often as patients in group B.

For group A patients, all dental procedures are postponed as far as possible until after the disease has been passed. If this is not possible, treatment is carried out in the clinic with the best possible protective equipment in a defined, separated section. To avoid contacts of the diseased person to other individuals, treatment in the patient’s quarantine area at home is considered in particular cases (e.g. opening of periodontal abscesses). Here, the dental team is supported by specialists in the treatment of viral diseases.

For the treatment of patients in groups B and C, the dental clinic was divided into 2 areas with separate entrances.

- One section is reserved exclusively for the patients of group C. In this area, a team that does not show any symptoms of disease or, if possible, is tested daily for SARS-CoV-2 before entering into service.
- The other section is reserved for group B and, if absolutely unavoidable for group A patients. If this division within a dental practice is not possible, practices in the neighbourhood should cooperate exclusively.

Our treatment teams of dental health care providers consist of three persons:

1. Experienced dentist in the contaminated area,
2. Experienced assistant in the contaminated area,
3. Second assistant in the non-contaminated area.

The second assistant ensures the supply of instruments, materials and equipment specifically required for the patient and assists in fitting and removing the protective clothing. Due to the high physical strain when working under complete protective equipment (disinfectable footwear, coverall/gown, surgeons hood, double gloves, individual magnifying glasses, N95 filtering respirator covered by surgical face mask, face shield covering the front and sides of the face), several teams are available depending on the number of patients. At least two additional personnel are required to guarantee the admittance and phone service as well as the cleaning and disinfection of the used treatment room.

The treatment rooms/dental units are operated in a rotation mode.

I. dental unit I is used for treatment,
II. unit II is cleaned and disinfected,
III. unit III is ready to be used for the treatment of the next patient.

Based on initial experience, the disinfection of a treatment room and the running of the complete disinfection program of the dental unit requires between 50 min and 1 h. This causes unit II to be blocked parallel to unit I for a certain period of time.

A typical operative dental treatment of a patient, e.g. preparation and placement of an extensive filling for a molar, under protective equipment takes more than 60 min. Here, the time for applying and removing the protective equipment is considered. After initial experiences, it has become clear that all preparatory steps and all treatment steps must be carried out calmly and carefully in the current situation. Every existing and new routine must be constantly re-evaluated.

The current situation therefore requires a fundamental re-thinking of treatment processes, staff and material resource planning. For each treatment team, three persons are required. Depending on the number of patients and due to the physical and psychological strain and the additional requirements for care, one or two additional teams must be planned. In addition, maximum efforts should be made to reduce the risk of vulnerable patients becoming infected with SARS-CoV-2 by dental professionals.

The other decisive limitation is the availability of treatment rooms or dental treatment units. It must be expected that even in larger practices with several treatment units, a much smaller number of patients can be treated daily.

It is therefore necessary to cooperate across single dental offices or to concentrate resources in larger practices or clinics.

## Clinical relevance

The approach described could help to ensure the effective use of dental care combined with the best possible protection of patients and staff. This will at least maintain the capacity for emergency dental treatment.
